# Supplementary material for: Heterotrimeric G Protein-Mediated Signaling Is Involved in Stress-Mediated Growth Inhibition in Arabidopsis thaliana
Source: Int J Mol Sci. 2023 Jul 3;24(13):11027. doi: 10.3390/ijms241311027 (PMC10342085; doi:10.3390/ijms241311027)
Supplement: Supplementary file 1 [file ijms-24-11027-s001.zip › ijms-2469809-supplementary.pdf]

Heterotrimeric G protein-mediated signaling is involved in stress-mediated growth inhibition in *Arabidopsis thaliana*

Soeun Yang, Seohee Jung and Horim Lee†

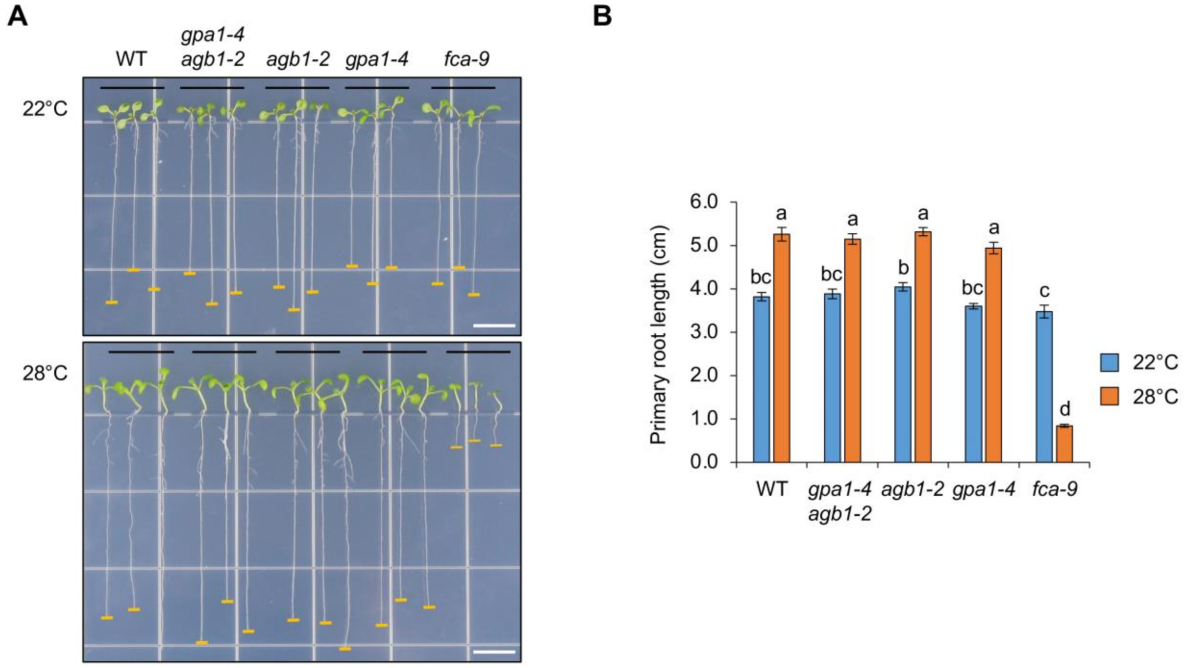

Figure S1. Heterotrimeric G protein mutants are not related to general thermomorphogenesis. (A) Growth phenotypes of WT Col-0, *gpa1-4 agb1-2*, *agb1-2*, *gpa1-4*, and *fca-9*. Seedlings were grown vertically on half MS plates under 22°C or 28°C conditions for 7 days. White scale bars, 1 cm. (B) Primary root length of seedlings grown under 22°C or 28°C conditions. The data are presented as the mean ± S.E. (n = 10~12). The different letters indicate a significant difference (p<0.05) according to the one-way ANOVA with post-hoc Turkey's HSD test.

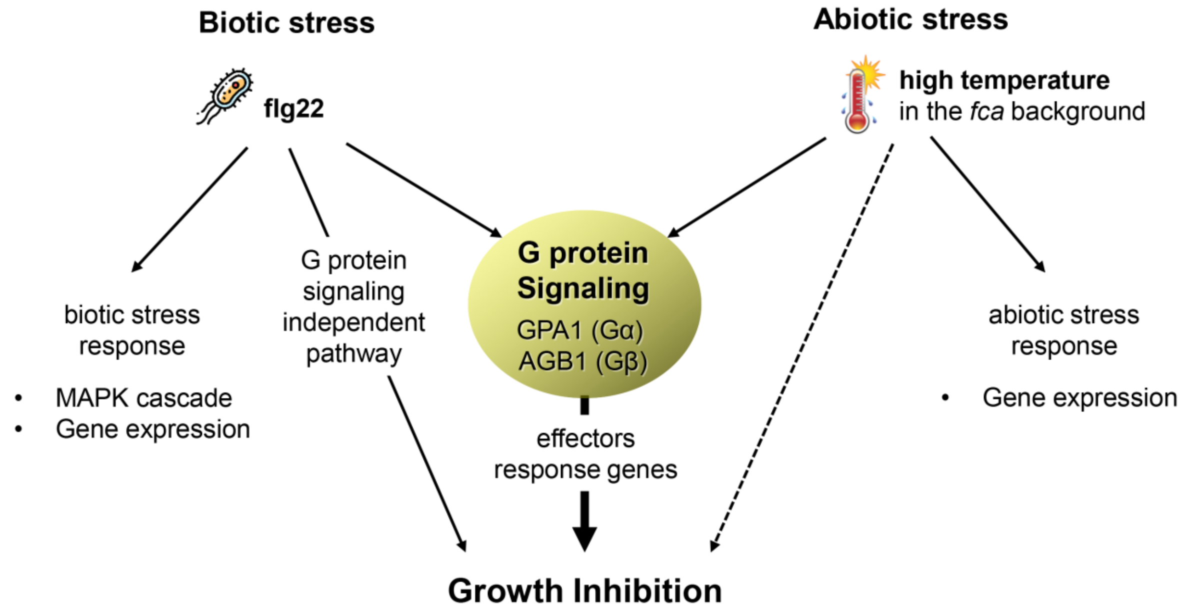

Figure S2. A scheme of G protein signaling-mediated growth inhibition in response to stress. Biotic stress such as flg22 triggers an immune response including MAPK activation, immune gene expression, and growth inhibition. Since growth inhibition, but not the biotic stress response, was suppressed in *agb1* by flg22 treatment, this suggests that growth inhibition is less tightly linked to early biotic stress responses via G protein signaling. As the phenotype rescued in *agb1* by flg22 was not complete, it also suggests that the G protein signaling-independent pathway for growth inhibition is involved. In addition, abiotic stress such as high temperature affects plant growth in the *fca* mutant background. The *FCA*-mediated growth inhibition was rescued in *gpa1*, suggesting that G protein signaling is involved in growth inhibition by abiotic stress conditions such as heat stress. A dotted line indicates the putative pathway that was not investigated in this study.

Table S1. Primers used in this study.

AGI	Primer	Sequence (5′—3′)
At4g34460	*agb1-2*_LP	CTGTACCGGTTTCAAGAATGCTC
	*agb1-2*_RP	CGTGTTGAAGGAGTTTAGTTCCC
	*agb1-2*_LB	GGCAATCAGCTGTTGCCCGTCTCACTGGTG
At2g26300	*gpa1-4*_LP	TAAAGCTTCGTTTATGCAGCC
	*gpa1-4*_RP	ATCGCTAAGTCTTTTGTCCCG
At4g16280	*fca-9*_forward	TGTGGAAGATGTCTATCTCATGCG
	*fca-9*_reverse	GTACCCAAGGCATTACCTTG
At3g18780	*qACT2*_forward	TCCCTCAGCACATTCCAGCAGA
	*qACT2*_reverse	AACGATTCCTGGACCTGCCTA
At5g24110	*qWRKY30*_forward	GTCAATGCCAAGGTGGAGTT
	*qWRKY30*_reverse	CTGGCCGTACTTTCTCCAAC
At1g18570	*qMYB51*_forward	TGGGCCAATTATCTTAGACCTG
	*qMYB51*_reverse	TGATCTCGTTATCGGTTCTTCC
At1g12820	*qAFB3*_forward	AGCTCGAGATGCTTTCGATAGCTTTTG
	*qAFB3*_reverse	TCATTCTGTTCCATCCCATTATTCTCA
At4g03400	*qGH3.10*_forward	AACCCGTCTCTCTTGCTTCTCA
	*qGH3.10*_reverse	CAACGTTCACACCAATCCAG
At2g43010	*qPIF4*_forward	AGATCATCTCCGACCGGTTT
	*qPIF4*_reverse	CGCCGGTGAACTAAATCTCA
